# The Role of Ozonated Jerusalem Artichoke Ointment on the Healing of Surgically Created Full-Thickness Cutaneous Wounds in Rabbits

**DOI:** 10.1155/vmi/9966943

**Published:** 2024-12-11

**Authors:** Ali Ghazi Atiyah, Mustafa Salah Hasan, Maher Saber Owain

**Affiliations:** ^1^Department of Internal Medicine, Surgery and Obstetrics, College of Veterinary Medicine, Tikrit University, Tikrit, Iraq; ^2^Department of Internal Medicine, College of Veterinary Medicine, University of Fallujah, Fallujah, Iraq

**Keywords:** Jerusalem artichoke, medicinal plants, ozone, wound healing

## Abstract

Jerusalem artichoke (J.A.) tubers contain compounds that exhibit anti-inflammatory effects and can minimize tissue damage. Ozone is an alternative antimicrobial and immunomodulatory agent for promoting tissue regeneration. The present study aimed to evaluate the therapeutic effect of the ozonated J.A. ointment on a surgically created full-thickness cutaneous wound in rabbit models. The previously prepared J.A. ointment was ozonated using a Herrmann generator, followed by a subsequent evaluation of its physical and antibacterial properties. Thirty healthy male albino rabbits were used in this study. The animals were divided into two equal groups: the control and treated group. An excisional wound model was used to assess wound healing activities. All of the animals underwent surgical preparation of their dorsal surfaces, and excisional lesions of 3 cm in diameter were created on each animal's dorsal surface of the thoracolumbar region. In the control group, the wounds were left untreated. The animals in the treatment group received a topical application of ozonated J.A. ointment twice daily for five days following the injury. The animals were euthanized on Days 7, 14, and 21 after the injury for histological evaluation. The agar well diffusion method demonstrated the antimicrobial efficacy of the ozonated J.A. ointment. Also, macroscopic and histopathological results showed a significant (*p* < 0.05) increase in wound area contraction with enhancement re-epithelization in the treated group compared to the control group. In conclusion, the ozonated ointment derived from J.A. tubers has antibacterial properties and can promote and enhance the wound healing process.

## 1. Introduction

Cutaneous wound healing is the process by which skin repairs itself following injury caused by surgery, trauma, or burns. The wound healing process involves the activation, proliferation, and movements of numerous cell types with different functions to restore the normal integrity of the skin through four specific stages of inflammation, proliferation, re-epithelialization, and remodeling stage [1]. The majority of wounds caused by surgery eventually heal, however, a small percentage get chronic wounds due to complications including infection or dehiscence. The majority of long-lasting wounds do not originate from surgical procedures, for example, 60,000 annual fatalities and 2 million annual hospital admissions are directly attributable to pressure ulcers [2]. Several studies are focused on developing more efficient wound treatments to decrease healthcare expenses and offer lasting relief, ultimately leading to effective scar healing. Skin wound remedies are categorized as either “conventional” or “regenerative.” Regardless of esthetic and potential functional changes, conventional treatment results in the development of scars. Regenerative wound therapy is an emerging field in biomedical research that focuses on restoring the function of the skin by repairing damaged cells and tissue without causing scarring (Boyce and Lalley, 2018; Fani et al., 2024).

The conventional wound medication or dressing materials seem to be insufficient to facilitate and support wound healing mechanisms [3]. The ideal wound medication has to give protection against bacterial infections without any adverse effects [4]. The overuse of antibiotics has led to antimicrobial resistance, making many drugs ineffective. Extensive research into the cellular mechanism of wound healing has been conducted on the numerous plant extracts that have recently been revealed to have wound healing activities [5]. The regenerative wound medication or dressing materials refer to the materials that are extracted from medicinal plants and have been reported to exhibit many biological activities such as antimicrobial, anti-inflammatory, and antioxidant activities [6]. There is some evidence that the antibacterial properties of medicinal plants work in ways that are distinct from the current antimicrobials already in use [7]. Furthermore, plant-based remedies are often less expensive than traditional treatments, and they are safer than synthetic alternatives [8].

Medicinal plants are rich in various natural chemical compounds, which have the potential effect on the healing process of wounds, and inhibit or reduce the inflammatory process of the wounds [9, 10]. The wound healing effects of medicinal plants are shown by the different mechanisms, such as modulation in the wound healing process, decreasing the bacterial count, improving deposition of the collagen fibers, and stimulation of fibroblasts and fibrocyte proliferation [11]. A member of the Heliantheae tribe, H. tuberosus L. is a member of the Asteraceae family and the Asteroideae subfamily. Annually, the H. tuberous plant grows thick, hairy stems that are filled with a spongy mass and are separated into nodes and internodes. Alternating with one other along the stem are the leaves. In addition to having a great resistance to pests and diseases, Jerusalem artichoke (J.A.) is characterized by its excellent tolerance to freezing temperatures and drought. It has a long history of cultivation as a medicinal herb and a highly prized food plant. For pain and broken bones, the leaves are a traditional remedy in folk medicine. In addition, the leaf components contain several bioactive chemicals with antifungal, antioxidant, and anticancer characteristics. This species' broad range of medicinal and pharmaceutical uses stems from its ability to reduce blood sugar levels as well as total cholesterol and triglycerides. It is an excellent substitute for conventional prebiotics as well [12]. J.A. has been scientifically demonstrated to enhance the process of wound healing by regulating inflammation, resulting in a more significant reduction in wound size and improved formation of wound epithelium. J.A. also can enhance wound healing by stimulating the growth and movement of fibroblasts and keratinocytes [13].

Ozone has recently made its way into the complementary medicine treatment of infected wounds caused by surgery or trauma. Ozone, a gas composed of three oxygen atoms, is intrinsically unstable. It has multiple potential uses, including in the transportation of oil, gas, and water [14]. Due to its antioxidant and highly reactive antimicrobial characteristics, ozone gas is widely used as a corresponding treatment for various skin disorders, such as diabetic foot, wound healing, bedsores, and other conditions, by reducing inflammation, increasing growth factor levels, further improving oxidative pressure state, and decreasing healing time [15]. The possible mechanisms of the topical application of ozone include promoting angiogenesis and tissue regeneration through increasing local oxygen levels and ATP transporter molecules in the damaged tissues. Also, ozone has a role in the immune system by inhibiting the migration of mast cells and reducing the release of some acute phase proteins and lysosomal enzymes [16]. Other recent studies suggest that the systemic use of O3 enhanced tissue healing of cutaneous wounds through its positive effects on cell differentiation and epithelial migration. This study demonstrated that when ozone reaches the cell membrane, it can activate the nuclear factor kappa-B (NF-*κ*B) pathways, consequently promoting the activation of intracellular pro- and anti-inflammatory cytokine pathways [17].

The present study was designed to evaluate the therapeutic effect of the topical use of ozonated J.A. ointment on a surgically created full-thickness cutaneous wound healing in rabbit models.

## 2. Materials and Methods

### 2.1. Ethical Approval

This study utilized the recommendations protocol of the Animal Care and Use Committee, at the College of Veterinary Medicine, Tikrit University, with approval license number (Tu. Vet.24) dated 11-5-2024.

### 2.2. Animals

Thirty male albino rabbits (6–8 weeks, weighing 150–200 g) were used to assess the wound healing activity. They were sourced from the animal house center at Tikrit University for the objectives of this study. They have been housed in sanitary conditions at standard temperature and humidity conditions (temperature: 25 ± 2°C, 55 ± 5% RH), with 12 h light/dark cycle. The animals were provided free-fed pellets and water from sanitary rodent-proof bottles and cleaned the cages, mattresses, and water bottles every day throughout the experiment.

### 2.3. Preparation of J.A. Ointment

**Figure 1:**
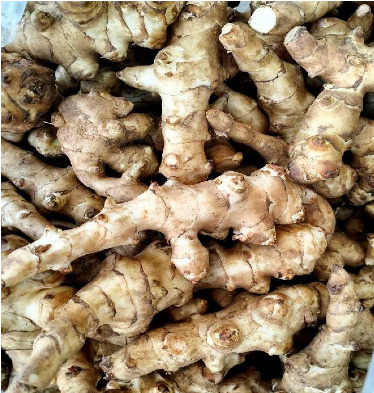
The J.A. tubers.

The J.A. tuber was bought in January and April 2023 at a market in Tikrit City, Iraq. Professor Dr. Rana H. Aloush AL-AL-Sammarai of the Department of Biology at the University of Tikrit in Iraq confirmed the plant's identity and authenticity (Figure 1). Using a slightly altered variation of a previously documented procedure, the J.A. ointment was prepared according to a modified method mentioned by the authors in [13]. Before being sliced longitudinally to obtain slices of about 2 mm thick, the plants were rinsed and dried under running tap water to remove any dirt or debris. A stainless-steel knife was then used for this purpose. Next, the samples were taken to the lab to undergo a freeze-drying procedure with the use of a lyophilizer (LG, China) to extract fine powder by removing moisture from the plants. Using a rotary mortar grinder (Retech, India), the dried J.A. slices were reduced to a fine powder with a size of 10 *μ*m. In order to make a 50% ointment, 50 g of the powder was mixed with 100 g of petroleum jelly that had been melted in a water bath at 65°C [18]. After a gentle mixing with magnetic starrier to ensure homogeneous mixing, the ointment base was cooled and then transferred to an appropriate container.

### 2.4. The Ozonation Process of J.A. Ointment

This study used the procedure outlined by the authors in [19] to make the ozonated J.A. ointment. First, 10% of previously piled J.A. ointment was exposed directly to a gas stream of O2/O3 with a concentration of 75 *μ*g/mL of ozone. This was performed under normal pressure conditions with a continuous flow of 4 L/min, using a Herrmann generator (Medozon Compact, Germany) to generate the ozone gas. The J.A. ointment was exposed to the gas stream for 3 h, resulting in a concentration of ozone of 17 mg/g of oil. The ozonated oil was then directly stored in a refrigerator at 4°C.

### 2.5. Physical Evaluation of Ozonated J.A. Ointments

#### 2.5.1. Determination of the PH of Ozonated J.A. Ointment

Before determining the PH of the ozonated J.A. ointment, the ointment was immersed in the water bath for 10 min to convert the ointment to the solution. Then, the pH value of the solution was found using a pH meter (AD1000, Germany). Following the manufacturer's instructions, the pH meter was operated. To begin, buffers with pH values of 4, 7, and 9 were used to calibrate the equipment. After that, the PH value was determined by submerging the electrodes directly in the solution.

### 2.6. Nonirritancy Test

An herbal ointment was applied to the skin of rabbits and observed for the effect.

### 2.7. Spreadability

In order to find the spreadability, an excess sample was crushed to a uniform thickness between two slides using a specific weight for a specific amount of time [20]. One way to test spreadability is by timing how long it took to separate the two slides. When the two slides are separated as quickly as possible, the spreadability improves. To determine spreadability, the following formula was used:

(1) $$\begin{array}{c} S=M\times \frac{L}{T}, \end{array}$$

where *S* is the spreadability, *M* is the weight tide to the upper slide, *L* is the length of the glass slide, and *T* is the time taken to separate the slides.

### 2.8. Antimicrobial Test

The sensitivity to antimicrobials was evaluated by using the Kirby–Bauer antibiogram technique. A bacterial culture suspension with a turbidity of 0.5 McFarland was obtained after the microorganisms, frozen at −20°C, were brought back to life. McFarland standards were used to achieve the desired turbidity in the bacterial suspensions. The 0.5 McFarland standard is used as the basis for the bacterial suspension of the organism to be evaluated in the Kirby–Bauer susceptibility test methodology. The Mueller–Hinton agar was used to transfer K. pneumonia and S. aureus cultures. The final volume of 75 *μ*L of ozonated J.A. ointment was in the middle of the plate. A zone of inhibition was seen after incubating at 37°C for 24 h. Antibiotic discs (Bioanalysis, Turkey) were used as a control for this study.

### 2.9. Surgical Wound Creation

All surgical procedures were conducted under strict aseptic conditions. The efficacy of the ozonated J.A. ointment in promoting wound healing was assessed by using an excisional wound model. The animals were sedated with a mixture of ketamine hydrochloride (35 mg/kg) and xylazine (10 mg/kg) administered through intramuscular injections [21]. Subsequently, the upper part of the back between the thoracic–lumbar regions was shaved and prepared for surgery, and the area of the skin to be removed was marked with a marker. Using a surgical knife (blade no. 11), circular excisional lesions measuring 3 cm in diameter were created along the previously defined area on all of the animals. Hemostasis was achieved by applying a sterile gauze soak after creating the wound. After inducing the excisional wounds, animals were divided equally into two groups. In the control group, the animals received no therapy. The treated group was topically treated with ozonated J.A. ointment twice daily for the first five days following the surgery.

### 2.10. Measurement of Wound Area Contraction

The variations in the wound area by contraction were measured on Days 0, 3, 7, 14, and 21 pos-surgeries. Measurement of the wound area contraction as a percentage was calculated with a caliper and using the following formula [22]:

(2) $$\begin{array}{c} \text{percentage of wound closure}=\left(\frac{\text{wound area zero}-\text{day}-\text{wound area day }\left(n\right)}{\text{wound area zero}-\text{day}}\right)\times 100, \end{array}$$

where *n* represents the 3^rd^, 7^th^, and 14^th^ days postwounding.

### 2.11. Macroscopic Evaluation

The macroscopical inspection of the wound's appearance, color, contraction, granulation tissue regularity, epithelization, and wound exudation was used to evaluate the wound healing quality at different intervals after injury. Digital photographs were taken of the wound area. The hair around the edges of the wound was clipped before taking the photos, and a measuring ruler was used as a standard for accurate measurements of wound contractions.

### 2.12. Histopathological Evaluation

The wound tissue samples were collected on Days 7, 14, and 21 after the injury. Five animal samples were taken from each group for histopathological investigation. The animals were terminated using a fatal dose administered through intramuscular injection of ketamine and xylazine. The full-thickness samples were removed using a combination of sharp and blunt scissors. They were then preserved in a solution of 10% buffered formalin, dehydrated with 70% alcohol, cleaned with xylene, and finally embedded in paraffin wax. The microtome (Leica, Germany) was used to create serial slices that were 5 *μ*m thick. These sections were then stained with hematoxylin and eosin. The pathologist examined the stained sections using a light microscope (Olympus, Japan).

### 2.13. Statistics Analysis

All statistical data are expressed as mean and standard deviation (SD). The statistical significance of differences between the two groups was analyzed when *p* was < 0.05 using an unpaired *t*-test. The statistical analyses were performed using GraphPad Prism (Version 8).

## 3. Results

The outcomes of the physicochemical evaluation parameters of ozonated J.A. ointment are presented in Table 1.

### 3.1. Macroscopic Evaluation

**Figure 2:**
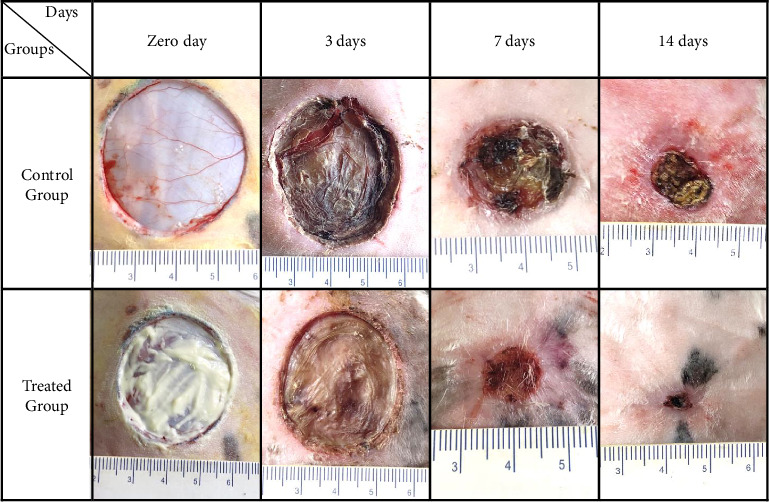
A macroscopic view of the treated and control groups shows the wound contractions taken at 0, 3, 7, and 14 days postsurgeries.

**Figure 3:**
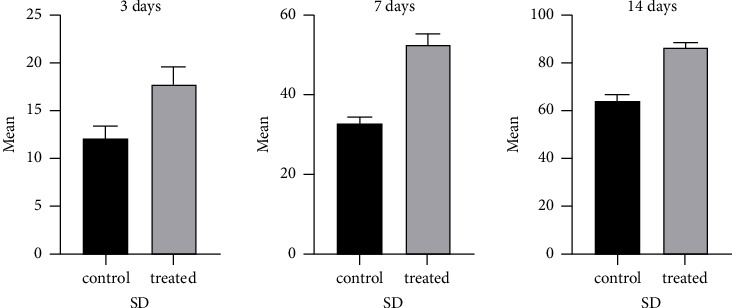
The mean and SD graphs of the wound contraction rats in both groups at 3, 7, and 14 days postsurgeries used unpaired *t*-tests at different interval days.

When examining the healing process of wounds, the control group displayed symptoms of inflammation, erythema, and edema within the first 7 days after injury. These symptoms persisted for up to 14 days and eventually led to signs of necrosis and excessive scab formation on Day 21. However, the treated did not exhibit any inflammation or erythema within the first 7 days after injury. By Day 14, the wound had contracted more rapidly and did not show obvious signs of necrosis or scab formation. By Day 21, the wound had nearly fully closed, as seen in Figure 2. The treated group had a significantly higher mean wound contraction (*p* < 0.05) than the control group, starting from Day 7 (84.43%) up to Day 21 (10.85%), as indicated in Figure 3.

Average measurements of wound contraction percentage at different interval periods in both groups are presented in Table 2.

### 3.2. Antimicrobial Activity of Ozonated J.A. Ointment

**Figure 4:**
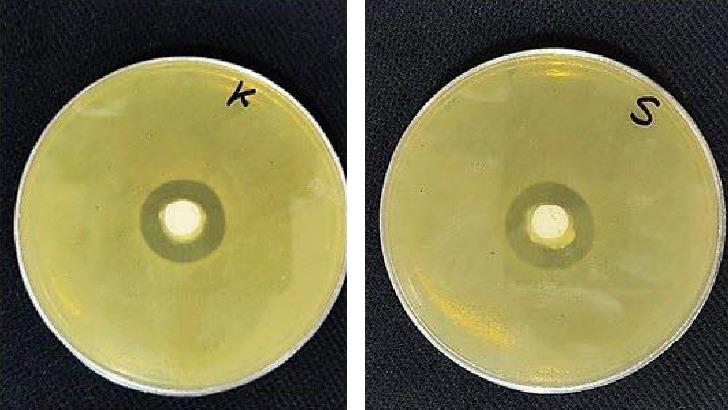
The zones of inhibition of ozonated J.A. ointment against (a) K*lebsiella pneumonia* and (b) *Staphylococcus aureus* used the Mueller–Hinton medium and incubated the mixture at 37°C for 48 h.

The antibacterial efficacy of ozonated J.A. ointment was assessed, as shown in Figure 4. The areas where the growth of different strains of *Klebsiella pneumonia* and *Staphylococcus aureus* bacteria appeared were clearly prevented.

### 3.3. Histopathological Evaluation

**Figure 5:**
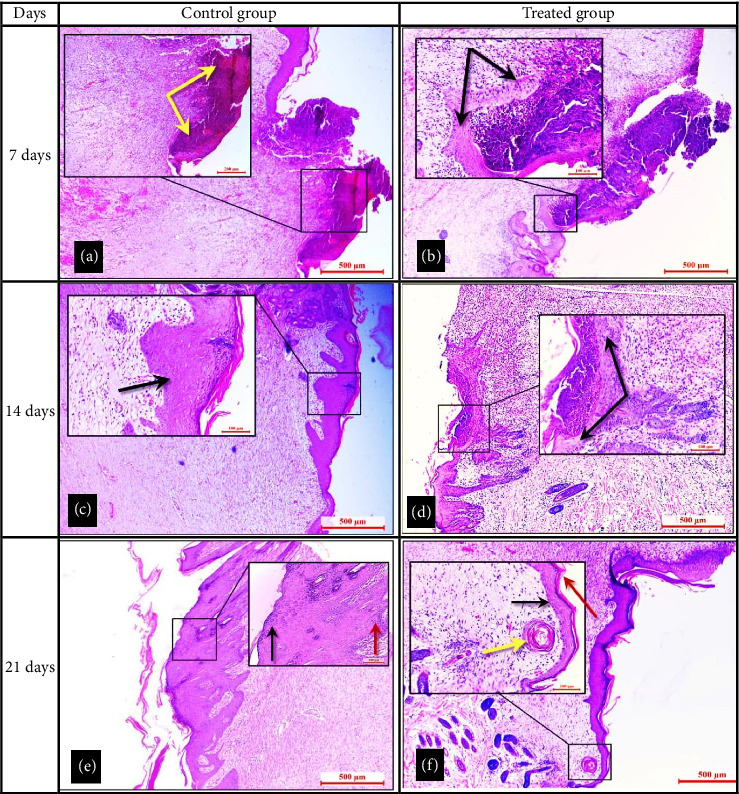
(a) The control group, seven days after injury, revealed a thick fibrin clot (yellow arrows) with absence of the development of the epidermal tongue. (b) The treated group, seven days after injury, observed well development of a thick epidermal tongue (black arrows) across the injury area. (c) The control group at 14 days showed a thick epidermal layer (epidermal hyperplasia) covering the injured area. (d) The treated group at 14 days after injury showed a complete re-epithelialization by a well-developed epithelial layer (black arrows). (e) The control group at 21 days after injury, observed hyperplasia of the thick epidermal layer marked by the development of a stratum granulosum layer (black arrow) with a stratum spinosum layer (red arrow). (f) The treated group at 21 days after surgery showed a well-organized epithelial layer (black arrow), a thin keratin layer (red arrow) covering the epidermis surface, and a newly formed hair follicle (yellow arrow) in the dermal layer. Hematoxylin and eosin staining, and the scale bar represents 200 and 500 μm.

**Figure 6:**
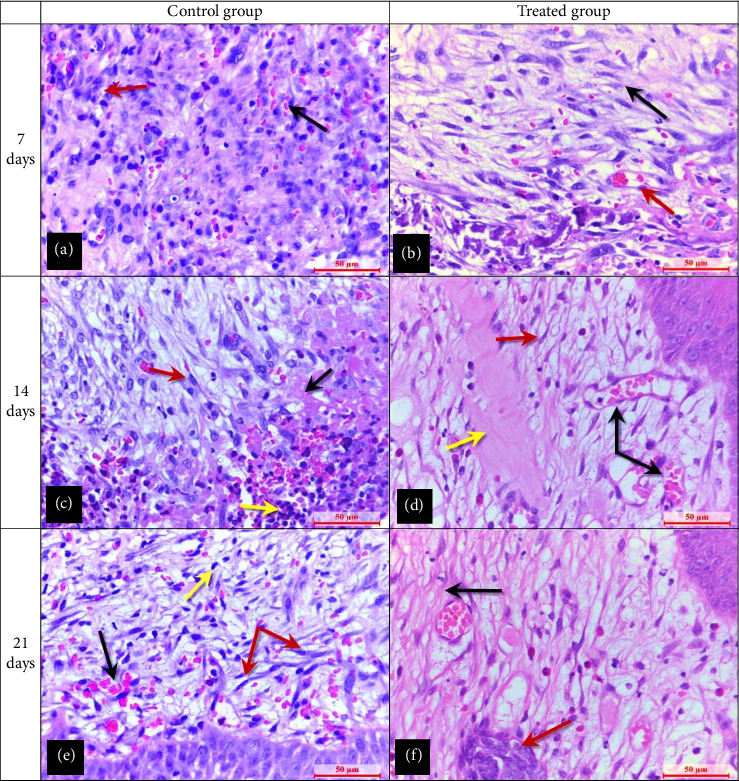
(a) Seven days postinjury, the control group exhibited hemorrhage (black arrow) and numerous multinucleated inflammatory cells in the dermal layer (red arrow). (b) The treated group at seven days showed many fibroblast cells (black arrow) with newly formed blood vessels (red arrow). (c) The control group at 14 days postinjury had extensive granulation tissue marked by inflammatory cells (yellow arrow) and fibroblasts (red arrow), with a dense collagen matrix (black arrow). (d) The treated group at 14 days showed mature fibroblasts (red arrow) and angiogenesis (black arrows), with a dense collagen matrix (yellow arrow). (e) At 21 days, the control group showed granulation tissue marked by fibroblasts (red arrows), inflammatory cells (yellow arrow), and visible hemostasis (black arrow). (f) The treated group at 21 days developed new sebaceous glands (red arrow) and mature regularly oriented dense collagen fibers (black arrow). Hematoxylin and eosin staining, and the scale bar represents 50 μm.

On the seventh day following the injury, the histopathological results showed that the control group had a thick fibrin clot, an incompletely formed epidermal tongue at the wound edge, and an injured area coated with a combination of fibrin and a thick serocellular crust (Figure 5(a)). Another section in the dermal layer showed a severe hemorrhage with inflammatory reactions through the infiltration of a large number of multinucleated inflammatory cells and fibrocytes and the presence of angiogenesis (Figure 6(a)). On Day 14 after injury, the development of thick epidermal layers with the presence of a large number of keratinocyte cells was observed (Figure 5(c)). Another section in the dermal layer showed profuse development of granulation tissue rich in multinucleated inflammatory cells and fibroblast cells were marked by the deposition of a dense collage matrix (Figure 6(c)). On Day 21 after injury, hyperplasia of the epidermal layer was observed, which was marked by the development of a thin layer of stratum granulosum layer with a thick stratum spinosum layer (Figure 5(e)). Another section in the dermal layer showed the dermal layer marked granulation tissue formation rich in fibroblast with irregular orientation dense collagen fibers, with obvious hemostasis (red blood cells) (Figure 6(e)).

The histopathological findings in the treated group on Day 7 after injury showed the epidermal layer infiltrated with multinucleated cells and a well-developed epithelial tongue in the dermal layer which was rich in differentiated epithelial cells appearing underlying the epidermal layer (Figure 5(b)). Another section in the dermal layer showed the development of the granulation tissue rich in multinucleated inflammatory cells and mature fibroblast cells with deposition of dense irregular orientation collagen fibers (Figure 6(b)). On Day 14 after injury, a complete development of the epithelial lounge (complete re-epithelialization by a thin layer of epithelium) was observed, which reaches from both edges of the wound (Figure 5(d)). Another section in the dermal layer showed a large number of mature fibroblast cells with angiogenesis and deposition of dense collagen matrix (Figure 6(d)). On Day 21 after injury, a complete epithelization surface with a thin keratin layer over the epidermis (Figure 5(f)) was observed. Another section in the dermal layer showed the start development of a newly formed sebaceous gland with the development of mature collage fibers that appeared as regular orientation (Figure 6(f)).

## 4. Discussion

The full-thickness cutaneous wounds take a long time to heal and are prone to infections and fluid loss, making the healing process even more challenging. Although substantial skin damage compromises the skin's structural and functional integrity, the assessment and wound care management of full-thickness cutaneous wounds are still challenging and still need the development of new and better methods to accelerate the cutaneous wound healing process [23]. Many studies have used medicinal plants for various objectives, but one common thread is their ability to speed up the healing process after a wound has occurred. This practice is common in both human and veterinary medicine [24, 25]. From a clinical point of view, the pH of the ozonated J.A. ointment lies in the normal pH range of living skin (6.8 ± 1). Thus, the ointment formulations did not produce any skin irritation, such as erythema and edema for about a week when applied over the skin. As a result, this formulation did not produce any skin irritation for about a week when applied over the skin. In addition, the ozonated medicinal ointment has a powerful bactericidal action against skin *Staphylococcus* and *Klebsiella* at the same PH level, and the antibacterial mechanism of the ozonated medicinal plant ointment is associated with the transformation of ozone into oxygen and a lone oxygen atom after undergoing breakdown. This atom acts as a radical, specifically targeting different structural components of numerous pathogenic microorganisms, such as bacteria and fungi [26, 27].

Moreover, wound closure between the treatment group and the control group on Days 0, 7, 14, and 21 after surgery revealed that the treated group exhibited a significantly reduced (*p* ≤ 0.05) wound contraction size and residual wound area compared to the control group. Therefore, these findings indicate that the application of ozonated J.A. ointment can improve the complete healing of full-thickness cutaneous wounds, as specifically, on Day 21, the treated group exhibited a substantial reduction in both wound size and residual wound area. Therefore, the application of ozonated J.A. on the skin's surface may impact the process of wound healing by influencing the regrowth of epithelial tissue in the wound area, rather than directly affecting the formation of blood clots or scabs, as shown in the control group. On the other hand, many studies found that J.A. tuber has many phytochemical compounds such as phenols, phenolic acids, flavonoids, isoflavones, and fatty acids that are responsible for promoting the healing of cutaneous wounds through maturation and differentiation of the damaged epithelial layer [13, 28].

According to recent research, when therapeutic amounts of ozone gas are applied locally to the damaged tissue, they have several beneficial effects. It starts with the generation of lipid oxidation, and reactive oxygen species, such as hydrogen peroxide (H_2_O_2_), are produced by this gas. This is followed by stimulation of the biological processes that support tissue regeneration after injury, such as vasodilatation, angiogenesis, and disinfectant action against most pathogens, as well as a release of some growth factors from platelets and endothelial cells [29]. However, the exact therapeutic mechanism of ozone gas on cutaneous wound healing is yet to be entirely understood.

The current histopathological results showed an increase in the number of dermal fibroblasts in the control group compared to the treated group. This result supported by the previous findings suggested the ability of ozone to induce expressions of PDGF and TGF-*β* from dermal fibroblast at the wound injury site [30, 31]. Thus, the current histopathological findings suggested that ozonated medicinal plant ointment could induce the proliferation and differentiation of fibroblasts, collagen fibers, and epithelial cells and it could affect the wound healing process. Many recent studies have shown that topical ozone can aid in wound healing by acting as a bioregulator that releases endothelial cellular factors when it comes into contact with biological fluids, decreasing inflammation, increasing angiogenesis, decreasing pain, and increasing local peripheral vasodilatation and oxygenation [16, 32]. According to recent studies, ozone can trigger the activation of the transcription factor NF-*κ*B signaling pathway, which plays a crucial role in controlling inflammatory reactions and, ultimately, the entire healing process of wounds [33–36].

Another study showed that when ozone comes in touch with a biological fluid, it releases endothelial cellular components, regulates the inflammatory phase, stimulates angiogenesis, increases local peripheral vasodilatation and consequent oxygenation, and avoids pain [37]. It seems to be a particularly popular therapy strategy in diabetic wound healing since it speeds the healing process and possesses antibacterial, immunomodulator, antioxidant, and oxygenation capabilities [38]. Besides, the J.A. tubers are rich in essential fatty acids such as palmitic acid, stearic acid, and myristic acid [13]. When applied topically to a cutaneous skin wound, the ozone molecule forms an ozonide compound by bonding between the double bonds of these fatty acids [35, 39]. This makes it suitable for the topical use of ozone to treat persistent infections on the skin and mucous membranes of the body. Furthermore, there are no reports about the toxic effect of ozonated ointment on the epithelium of the cutaneous wound nor side effects on the wound healing process [40, 41].

In addition, it has been demonstrated that ozone can enhance the phosphorylation of the insulin-like growth factor 1 receptor (IGF1R), the epidermal growth factor receptor (EGFR), and the receptors for vascular endothelial growth factor (VEGFR). This further supports the potential therapeutic use of ozone on chronic wounds [36]. Thus, due to the high affinity between the phytochemical compound of J.A., especially the polyunsaturated fatty acids and the ozone, the biological synergism between the ozone and J.A. has a beneficial value for accelerating the physiochemical process of wound healing through a complex process, primarily involving ozonolysis, when ozone selects the polyunsaturated fatty acids as a preferred substrate to generate H_2_O_2_ within the first few minutes. While it is partly quenched by antioxidants, electrons are donated to ozone by surrounded polyunsaturated fatty acids. After that, an H2O2 gradient is established between the plasma and the cytoplasmatic membrane of living cells. The establishment of an H_2_O_2_ gradient between plasma and cytoplasmatic membrane of blood cells makes this oxidant a very early effector. This then quickly passes through the cell membrane, leading to the activation of several relevant biochemical signaling pathways within the cells such as activating the pentose cycle in erythrocytes, a tyrosine kinase in lymphocytes, and the release of growth factors from platelets and inflammatory cells [42, 43].

In addition, when ozone molecules rapidly react with fatty acids of the medicinal plant, the gas in these substrates changes into its active form and creates a 1,2,4-trioxolane structure [44]; this structure is the stabilized oxygen molecules' ring in the ozonized plant matrices and can slowly release ozone gas on the skin tissue, which can improve immunological functions through secondary metabolites called ozonides and can enhance autophagy, a cellular mechanism that aids innate immunity in the elimination of damaged organelles and intracellular infections, and as a result, speeds up the healing process through the enhanced proliferation of fibroblast and epidermal cells and subsequent increased production of Type-I collagen [45–47]. Also, the current histopathological sections showed that the angiogenesis process appeared throughout the newly formed blood vessels in the treated group compared to the control group. Thus, the current study agrees with the recent work that showed that the application of topical ozonated medium on the skin wound can improve vascularization at the wound leading edge in mice [15, 48].

Ozone exerts a regulatory influence on the phytochemical compounds, primarily affecting flavonoid production and phenylalanine metabolism in response to ozone exposure, serving as a crucial regulator of cellular antioxidant capability. Recent investigations indicate that optimal ozone synergism with phytochemical substances can markedly enhance peroxidase activity, suppress polyphenol oxidase activity, and preserve elevated levels of total phenols and flavonoids. These findings offer novel insights into the impact of ozone on phytochemical components and will assist in developing therapeutic medications [27]. Consequently, the current clinical results showed no adverse effects in the short-term observation period, but the long-term safety profile of ozonated ointments is not well-established. Thus, a more exact mechanism of action on how ozone works at the cellular and molecular level is needed.

## 5. Conclusion

The current study demonstrates that ozonated J.A. ointment exhibits synergistic effects by combining the therapeutic properties of ozone and J.A., which enhance its antibacterial activity and accelerate wound healing. Specifically, the ointment improved re-epithelization and promoted better collagen deposition in surgically induced full-thickness cutaneous wounds in rabbits. These findings suggest that ozonated J.A. ointment may be a promising therapeutic option for enhancing wound healing in clinical settings. Future research should focus on expanding the scope of this study by investigating the long-term effects of ozonated J.A. ointment, including potential side effects such as inflammatory responses or toxicity associated with prolonged use. In addition, exploring the use of other ozonated medicinal plant tubers, such as ginger, could provide valuable insights into the broader applicability of ozonated plant-based treatments for wound healing.

## Figures and Tables

**Table 1:** Physicochemical evaluation of the formulated ointment.

Physical evaluation parameters	Observation
Color	Yellow
Odor	Aromatic
Consistency	Smooth
PH	5.5
Spreadability	6 cm
Nonirritancy test	Nonirritant

**Table 2:** Average measurements of wound contraction percentage at different interval periods in both groups.

Values	3 days		7 days		14 days	
	Control group	Treated group	Control group	Treated group	Control group	Treated group
Mean	12%	18%	33%	52%	65%	87%
Standard deviation	1.33%	1.8%	1.6%	2.6%	2.4%	1.8
*t*-value	8.1^∗^		20.4^∗^		23.5^∗^	

^∗^ *p* ≤ 0.05.

## Data Availability

The data that support the findings of this study are available on request from the corresponding author. The data are not publicly available due to privacy or ethical restrictions.

## References

[1] Albahri G., Badran A., Hijazi A. (2023). The Therapeutic Wound Healing Bioactivities of Various Medicinal Plants. *Life*.

[2] Dehkordi A., Borojeny L., Albatineh A., Gheshlagh R. (2020). The Incidence of Pressure Ulcers and Its Associations in Different Wards of the Hospital: A Systematic Review and Meta-Analysis. *International Journal of Preventive Medicine*.

[3] Malabadi R., Kolkar K., Acharya M., Nityasree B., Chalannavar R. (2022). Wound Healing: Role of Traditional Herbal Medicine Treatment. *IJISRR*.

[4] Kalelkar P., Riddick M., García A. J. (2021). Biomaterial-Based Antimicrobial Therapies for the Treatment of Bacterial Infections. *Nature Review Materials*.

[5] Firdous S. M., Sautya D. (2018). Medicinal Plants With Wound Healing Potential. *Bangladesh Journal of Pharmacology*.

[6] Vaou N., Stavropoulou E., Voidarou C., Tsigalou C., Bezirtzoglou E. (2021). Towards Advances in Medicinal Plant Antimicrobial Activity: A Review Study on Challenges and Future Perspectives. *Microorganisms*.

[7] Vaou N., Stavropoulou E., Voidarou C., Tsigalou C., Bezirtzoglou E. (2021). Towards Advances in Medicinal Plant Antimicrobial Activity: A Review Study on Challenges and Future Perspectives. *Microorganisms*.

[8] Monika P., Chandraprabha M. N., Rangarajan A., Waiker P. V., Chidambara Murthy K. N. (2021). Challenges in Healing Wound: Role of Complementary and Alternative Medicine. *Frontiers in Nutrition*.

[9] Anand U., Jacobo-Herrera N., Altemimi A., Lakhssassi N. (2019). A Comprehensive Review on Medicinal Plants as Antimicrobial Therapeutics: Potential Avenues of Biocompatible Drug Discovery. *Metabolites*.

[10] Ghuman S., Ncube B., Finnie J. (2019). Antioxidant, Anti-Inflammatory and Wound Healing Properties of Medicinal Plant Extracts Used to Treat Wounds and Dermatological Disorders. *South African Journal of Botany*.

[11] Farahpour M. (2019). Medicinal Plants in Wound Healing. *Wound Healing: A Cellular Perspective*.

[12] Sawicka B., Skiba D., PszczóÅ‚kowski P., Aslan I., SharifiRad J., Krochmal-Marczak B. (2020). Jerusalem Artichoke (Helianthus Tuberosus L.) as a Medicinal Plant and Its Natural Products. *Cellular and Molecular Biology*.

[13] Atiyah A., Al-Falahi N. (2021). The Role of Helianthus Tuberosus Powder in Healing of Full-Thickness Wounds in Mice. *Veterinary World*.

[14] Varghese L. J., Lahiri B., Penumatsa N. V., Soans C. R., Sekar A., Nasyam F. A. (2024). Effectiveness of Topical Ozone Gel Application in the Management of Postextraction Wound Healing: An In Vivo Study. *The Journal of Contemporary Dental Practice*.

[15] Rizky A., Tanjung D., Khairunnisa K. (2024). The Effect of Ozone Therapy Stimulation on Diabetes Wound Healing Process. *Contagion: Scientific Periodical Journal of Public Health and Coastal Health*.

[16] Pivotto A., de Souza Lima L., Michelon A. (2024). Topical Application of Ozonated Sunflower Oil Accelerates the Healing of Lesions of Cutaneous Leishmaniasis in Mice Under Meglumine Antimoniate Treatment. *Medical Microbiology and Immunology*.

[17] Medeiros Cardoso d., Edilson Ervolino j., Massamitsu Miyasawa E. (2024). Unveiling the Therapeutic Potential of Systemic Ozone on Skin Wound Repair: Clinical, Histological, and Immunohistochemical Study in Rats. *BioMed Research International*.

[18] Yassine K., Houari H., Mokhtar B., Karim A., Hadjer S., Imane B. (2020). A Topical Ointment Formulation Containing Leaves’ Powder of Lawsonia Inermis Accelerate Excision Wound Healing in Wistar Rats. *Veterinary World*.

[19] Silva V., Peirone C., Capita R. (2021). Topical Application of Ozonated Oils for the Treatment of MRSA Skin Infection in an Animal Model of Infected Ulcer. *Biology*.

[20] Suruse P., Jadhav B., Barde G. (2023). Exploring the Potential of Aerva Lanata Extract in a Herbal Ointment for Fungal Infection Treatment. *Journal of Survey in Fisheries Sciences*.

[21] Hassan M. A., Abdalla M. A., Saber M. S., Senosy W., Nassif M., ElSherif M. W. (2024). Evaluation of Xylazine Ketamine Anesthesia in Rabbits Undergoing Tendon Surgery: A Prospective Randomized Controlled Study. *New Valley Veterinary Journal*.

[22] Teoh S., Latiff A., Das S. (2009). The Effect of Topical Extract of Momordica Charantia (Bitter Gourd) on Wound Healing in Nondiabetic Rats and in Rats With Diabetes Induced by Streptozotocin. *Clinical and Experimental Dermatology*.

[23] Hosseini M., Shafiee A. (2021). Engineering Bioactive Scaffolds for Skin Regeneration. *Small*.

[24] de Cantuário Ferreira P. G., Inocêncio Cunha L., Gomes Rabelo P. (2020). Use of Phytotherapics, Low Power Laser and Ozone for Biting Wound in Dog. *Acta Scientiae Veterinariae*.

[25] Sun W., Shahrajabian M. H. (2023). Therapeutic Potential of Phenolic Compounds in Medicinal Plants Natural Health Products for Human Health. *Molecules*.

[26] Travagli V., Zanardi I., Bocci V. (2009). Topical Applications of Ozone and Ozonated Oils as Anti-Infective Agents: An Insight Into the Patent Claims. *Recent Patents on Anti-Infective Drug Discovery*.

[27] Wang X., Liao D., Ji Q. (2022). Analysis of Bactericidal Effect of Three Medical Ozonation Dosage Forms on Multidrug-Resistant Bacteria From Burn Patients. *Infection and Drug Resistance*.

[28] Alemu M., Lulekal E., Asfaw Z. (2024). Antibacterial Activity and Phytochemical Screening of Traditional Medicinal Plants Most Preferred for Treating Infectious Diseases in Habru District, North Wollo Zone, Amhara Region, Ethiopia. *PLoS One*.

[29] Pasek J., Szajkowski S., Cieślar G. (2024). Effect of Treatment of Neuropathic and Ischemic Diabetic Foot Ulcers With the Use of Local Ozone Therapy Procedures—An Observational Single Center Study. *Clinical Practice*.

[30] Kim H., Noh S., Han Y. (2009). Therapeutic Effects of Topical Application of Ozone on Acute Cutaneous Wound Healing. *Journal of Korean Medical Science*.

[31] Prajoko Y. W., Manapa C. H., Nugroho T. N. (2024). Ozonated Aloe Vera Oil Accelerates Radiation Dermatitis Healing in Sprague Dawley Rats. *Middle East Journal of Cancer*.

[32] Özaydin I., Aksoy Ö., Yayla S. (2018). Clinical, Histopathological and Immunohistochemical Evaluation of the Effects of Topical NPH-Insulin on Full-Thickness Open Wounds: An In Vivo Study in Diabetic and Non-Diabetic Mice. *Ankara Universitesi Veteriner Fakultesi Dergisi*.

[33] Zeng J., Lei L., Zeng Q. (2020). Ozone Therapy Attenuates NF-Κb-Mediated Local Inflammatory Response and Activation of Th17 Cells in Treatment for Psoriasis. *International Journal of Biological Sciences*.

[34] Liu L., Zeng L., Gao L., Zeng J., Lu J. (2023). Ozone Therapy for Skin Diseases: Cellular and Molecular Mechanisms. *International Wound Journal*.

[35] Sallustio F., Fiorentino M., Pontrelli P. (2024). Ozone Therapy in Wound Care. *Pearls and Pitfalls in Skin Ulcer Management*.

[36] Franzini M., Valdenassi L., Pandolfi S., Tirelli U., Ricevuti G., Chirumbolo S. (2023). The Role of Ozone as an Nrf2-Keap1-ARE Activator in the Anti-Microbial Activity and Immunity Modulation of Infected Wounds. *Antioxidants*.

[37] Ugazio E., Tullio V., Binello A., Tagliapietra S., Dosio F. (2020). Ozonated Oils as Antimicrobial Systems in Topical Applications. Their Characterization, Current Applications, and Advances in Improved Delivery Techniques. *Molecules*.

[38] Anzolin A., da Silveira-Kaross N., Bertol C. (2020). Ozonated Oil in Wound Healing: What Has Already Been Proven?. *Medical Gas Research*.

[39] Valacchi G., Fortino V., Bocci V. (2005). The Dual Action of Ozone on the Skin. *British Journal of Dermatology*.

[40] Tonus S., Bayıl Oğuzkan S., Uğraş H., Kıılıç İ. (2018). Determining the Cytotoxic Effect Potential of Ozonated Hazelnut Oil. *Ozone Therapy*.

[41] Radzimierska-Kaźmierczak M., Śmigielski K., Sikora M. (2021). Olive Oil With Ozone-Modified Properties and Its Application. *Molecules*.

[42] Bocci V., Zanardi I., Michaeli D., Travagli V. (2009). Mechanisms of Action and Chemical-Biological Interactions Between Ozone and Body Compartments: A Critical Appraisal of the Different Administration Routes. *Current Drug Therapy*.

[43] Alp A., Polat E., Yenigun A., Pasin O., Ozturan O. (2024). Effect of Medical Ozone Therapy in Preventing Compromised Nasal Skin in Revision Rhinoplasty. *Aesthetic Plastic Surgery*.

[44] Puxeddu S., Scano A., Scorciapino M. (2024). Physico-Chemical Investigation and Antimicrobial Efficacy of Ozonated Oils: The Case Study of Commercial Ozonated Olive and Sunflower Seed Refined Oils. *Molecules*.

[45] Baldo M. E., Alves G. R., Saliba C. L. d. S., Ramalho R. T., Cury E. R. J. (2024). The Use of Ozonized Oil as a Therapeutic Conduct for Skin Lesions. *Contribuciones a las Ciencias Sociales*.

[46] Farid Z. S., Habba D. A., Balbola G. A., Bakr N. M. (2024). Periodontal Regeneration Related to Periodontitis Treated by Garden Cress and Ozone Therapy (Randomized Clinical and Experimental Study). *Journal of Medicine in Scientific Research*.

[47] Tang Z., Hu B., Zang F., Wang J., Zhang X., Chen H. (2019). Nrf2 Drives Oxidative Stress-Induced Autophagy in Nucleus Pulposus Cells via a Keap1/Nrf2/p62 Feedback Loop to Protect Intervertebral Disc From Degeneration. *Cell Death and Disease*.

[48] Lu J. Y., Wang X., Fu Z. (2023). Topical Ozone Accelerates Diabetic Wound Healing by Promoting Re-Epithelialization Through the Activation of IGF1R–EGFR Signaling. *Journal of Investigative Dermatology*.

